# Current status, challenges, and integration pathways of biomarker classification systems in graft-versus-host disease: a preliminary exploration

**DOI:** 10.3389/fmed.2025.1690221

**Published:** 2025-10-31

**Authors:** Xinxin Yu, Shuai Huang, Xiaoxia Li, Yizhuo Zhao, Xiaohan Jin, Meiqi Fan, Yuanfeng Zhang, Lusheng Ma

**Affiliations:** ^1^School of Clinical Medicine, Shandong Second Medical University, Weifang, China; ^2^Department of Ophthalmology, Yantai Yuhuangding Hospital, Yantai, China; ^3^Second Clinical Medical College, Binzhou Medical University, Yantai, China; ^4^Qingdao University Qingdao Medical College, Qingdao, Shandong, China; ^5^Department of Hematology, Yantai Yuhuangding Hospital, Yantai, China

**Keywords:** graft-versus-host disease, biomarker, classification system, integration pathways, allogeneic hematopoietic stem cell transplantation, precision medicine

## Abstract

**Background:**

Graft-versus-host disease (GVHD) is a life-threatening complication of allogeneic hematopoietic stem cell transplantation that impairs clinical outcomes. Existing classification systems for GVHD biomarkers remain fragmented, which limits cross-study data integration and clinical translation, creating an urgent need for a systematic classification framework.

**Materials and methods:**

In this review, a predefined search strategy was used to systematically evaluate the classification systems of GVHD biomarkers. For the search, a systematic literature retrieval was conducted in the PubMed and Web of Science databases, covering the time range from 2012 to 2025, with keywords including “GVHD,” “biomarkers,” and “classification and summarization.” The inclusion criteria for studies were as follows, focusing on the classification or clinical application of GVHD biomarkers: peer-reviewed original articles, reviews, or multicenter trials, and human subjects or well-validated mouse models. After screening, a total of 139 articles were included in this review.

**Conclusion:**

This review integrates GVHD biomarkers into a three-dimensional system, including pathophysiological mechanisms, clinical application scenarios, and molecular characteristics. It identifies key challenges in biomarker research and application, and proposes feasible integration pathways. This work provides a foundational framework for precision medicine in GVHD management.

## 1 Introduction

Allogeneic hematopoietic stem cell transplantation (allo-HSCT) usually serves as a primary therapeutic modality for hematological diseases; however, graft-versus-host disease (GVHD) remains a major barrier to improving treatment outcomes (1). Biomarkers, which function as indicators of normal physiological processes, pathological conditions, or responses to interventions such as exposure, are widely applied in disease diagnosis, monitoring, and the development of therapeutic approaches (2). They have become critical tools for guiding GVHD diagnosis, prognostic stratification, and treatment response monitoring, yet inconsistencies in their classification severely compromise clinical utility (3). For instance, interleukin-6 (IL-6) is defined as an inflammation-driven marker in preclinical mechanistic studies of GVHD; in clinical cohort studies, however, it is classified as a diagnostic marker for ocular graft-versus-host disease (oGVHD)—such classification discrepancies impede the integration of cross-study data.

Although numerous reviews on GVHD biomarkers have been published, most focus on individual molecules or single application scenarios and fail to address the core issue of “fragmented classification” by proposing targeted solutions. This research gap limits the translation of biomarker research into standardized clinical practice, as clinicians lack a unified framework for interpreting and applying these biomarkers (4). Therefore, by systematically synthesizing existing evidence, the present review aims to describe a coherent, multi-dimensional classification system for GVHD biomarkers, with the goal of resolving classification inconsistencies and facilitating their reliable application in clinical decision-making.

## 2 An overview of the pathogenesis of GVHD

### 2.1 Acute GVHD (aGVHD): innate immunity-driven inflammatory cascade

**Figure 1:**
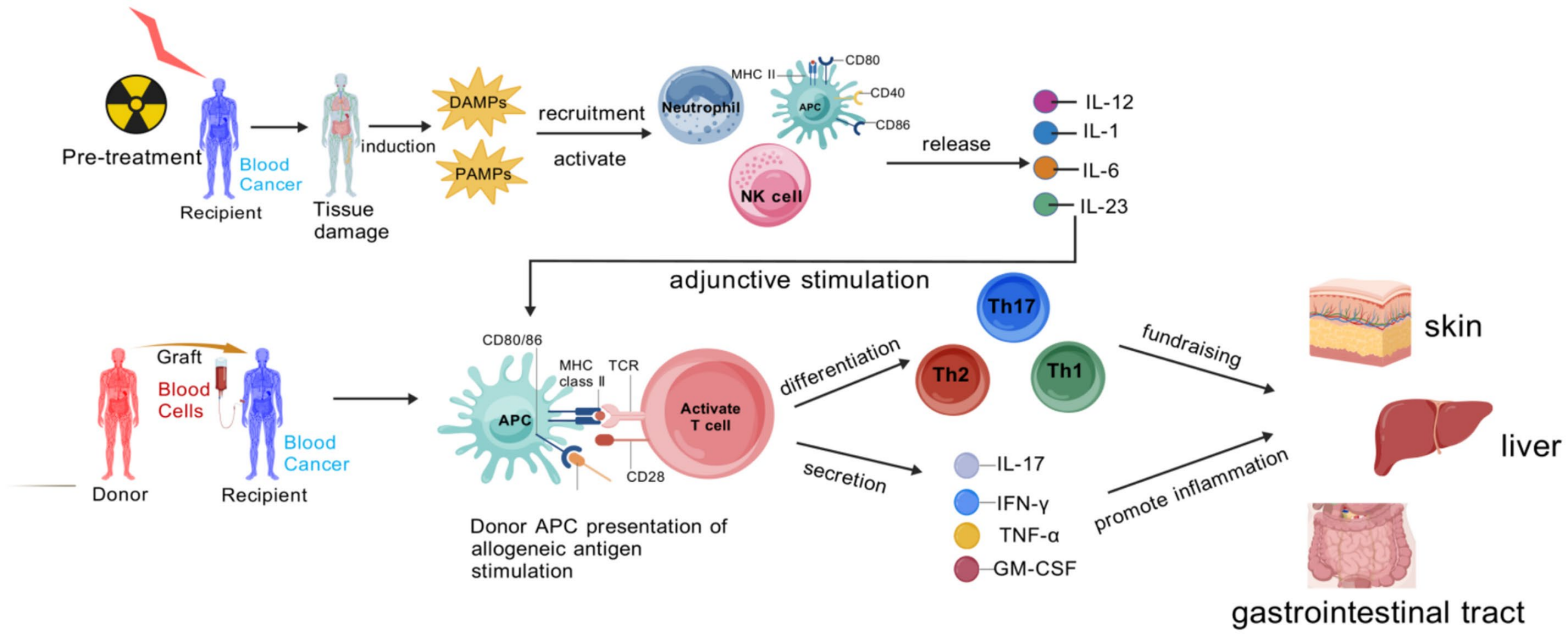
Pathogenic cascade of aGVHD.

The pathogenic process of aGVHD is centered on a three-stage “initiation-activation-effector” cascade, which proceeds as follows (5) (Figure 1):

Initiation stage: pretransplant conditioning induces tissue damage in recipients, triggering the release of damage-associated molecular patterns (DAMPs) (6) that subsequently activate antigen-presenting cells (APCs).
Activation stage: activated APCs present recipient alloantigens, which drive the differentiation of donor naive T cells into effector T cell subsets, such as T-helper 1 (Th1) and T-helper 17 (Th17) cells. Notably, interleukin-12 (IL-12) secreted by APCs during this stage acts as a biomarker for early inflammatory activation in aGVHD.
Effector stage: effector T cells, along with cytokines interferon-*γ* (IFN-γ) (7), tumor necrosis factor-*α* (TNF-α), secrete and infiltrate target organs, including the intestine, skin, and liver, and mediate tissue damage. Among these, regenerating islet-derived protein 3α (REG3α)—which is elevated upon intestinal epithelial injury—and Elafin—associated with skin injury—serve as specific tissue damage biomarkers for intestinal and cutaneous involvement in aGVHD, respectively.

The stage-specific immune drivers of aGVHD primarily revolve around the TNF-*α*/IL-1/IL-6 axis. During the initiation phase, TNF-α and IL-1 are produced by activated innate immune cells, which not only induce local inflammatory responses but also promote immune cell recruitment by upregulating the expression of vascular endothelial cell adhesion molecules (8). IL-6 exhibits pleiotropic effects: it not only promotes T cell proliferation and differentiation but also participates in the synthesis of hepatic acute-phase proteins, exacerbating systemic inflammatory responses. In the activation and effector phases, sustained high expression of these cytokines maintains a “cytokine storm,” further activating effector T cells and enhancing their cytotoxic activity, while exacerbating damage to vascular endothelial cells and target organ cells, leading to tissue necrosis (9).

Pre-transplant conditioning in recipients eradicates malignant cells but also inflicts damage on normal tissues, triggering the release of DAMPs and PAMPs. These molecular patterns mobilize and activate neutrophils, natural killer (NK) cells, and APCs. Once activated, APCs enhance the expression of MHC class II and co-stimulatory molecules, and secrete pro-inflammatory cytokines such as IL-1, IL-6, IL-12, and IL-23, which serve as essential adjuvant signals for T cell activation. Subsequently, donor-derived APCs recognize recipient alloantigens, and through the interaction between MHC class II and T cell receptors (TCRs) during antigen presentation, naive T cells are activated. These activated T cells then differentiate into Th1, Th2, and Th17 subsets, releasing cytokines like IFN-*γ*, TNF-*α*, IL-17, and GM-CSF. These cytokines orchestrate inflammatory reactions that ultimately cause tissue injury in target organs. Created with BioGDP.com (10).

### 2.2 cGVHD: adaptive immune dysregulation and fibrotic remodeling

**Figure 2:**
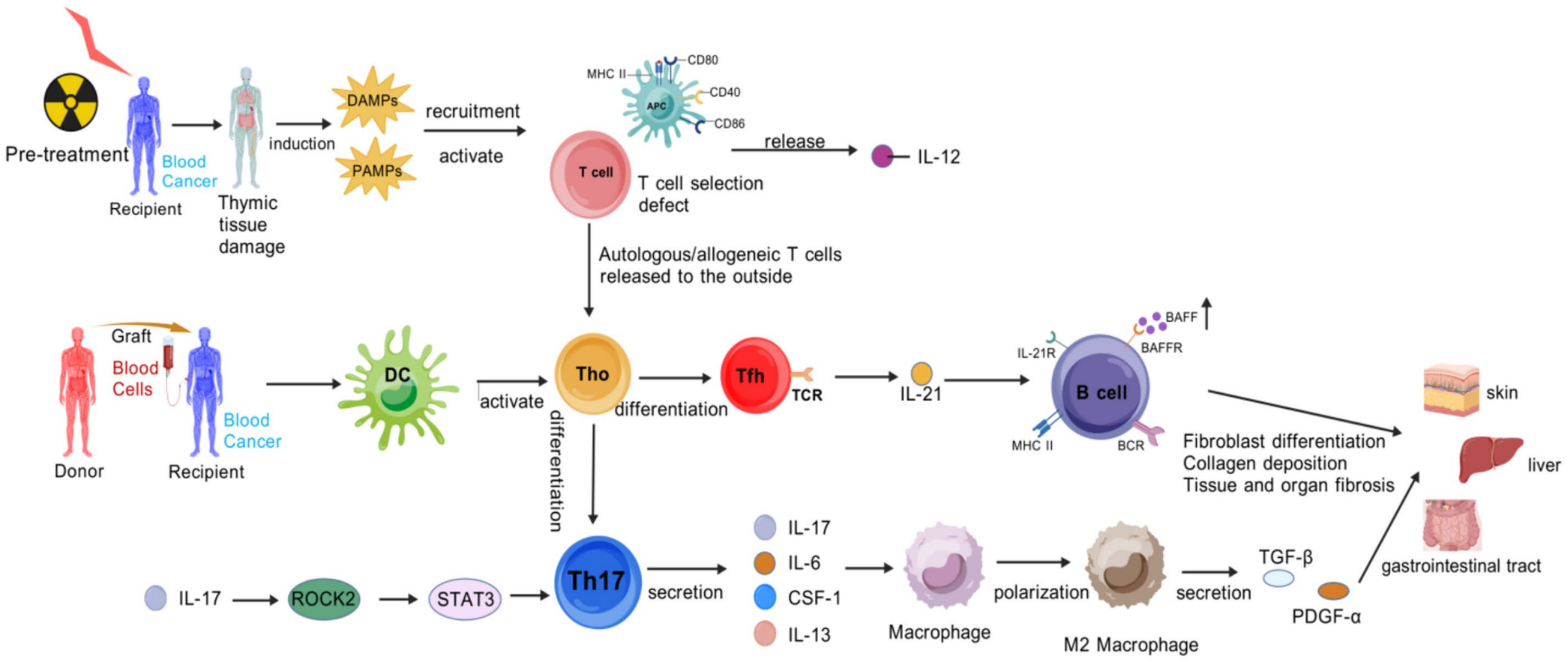
Pathogenic cascade of cGVHD. Pre-transplant conditioning damages the thymus, impairing negative selection of T cells. Autoreactive T cells evade deletion and enter the periphery. Concurrent tissue injury releases DAMPs, and gut microbial translocation releases PAMPs, APCs that secrete IL-12. Donor-derived dendritic cells (DCs) present auto−/alloantigens to naive T cells (Th0), driving their differentiation into two pathogenic subsets: Th17 cells. An autocrine loop via IL-17 → ROCK2 → STAT3 signaling amplifies Th17 differentiation. Th17 secretes cytokines (IL-17, IL-6, CSF-1, IL-13) to recruit and polarize macrophages toward a profibrotic M2 phenotype. Follicular helper T (Tfh) cells: Express TCR and secrete IL-21, which activates B cells through IL-21R, BAFF/BAFFR, and MHC-II/BCR interactions. M2 macrophages secrete TGF-β and PDGF-α, inducing fibroblast differentiation and collagen deposition, leading to fibrosis in target organs. Activated B cells produce autoantibodies and perpetuate T cell activation via antigen presentation, forming a pathogenic feedback loop. Created with BioGDP.com (10).

Chronic graft-versus-host disease (cGVHD) is characterized by immune homeostasis dysregulation and tissue fibrotic remodeling as key features. Its disease course progresses in three phases (11) (Figure 2).

Early Inflammation and Tissue Injury: Tissue damage induced by transplantation preconditioning releases DAMP/PAMP, which activates APCs to upregulate MHC class II molecules and costimulatory molecules, and secrete IL-12. Donor T cells differentiate into Th1/Th17 subsets under the action of IL-6 and TGF-β, and secrete IL-17, IFN-*γ*, and IL-17 exacerbate epithelial/endothelial damage, thereby forming a proinflammatory microenvironment. IL-12, IL-6, and other factors are not only regulatory factors, but also inflammation-related biomarkers (12).

Chronic Inflammation and Immunodysregulation: it is centered on abnormal B-cell activation and impaired regulatory T-cell (Treg) function—B cells overproduce autoantibodies and rely on B-cell activating factor (BAFF) for survival, and elevated BAFF levels are closely associated with cGVHD activity (13).

Fibrosis and Tissue Remodeling (14): Myofibroblasts highly express *α*-smooth muscle actin (α-SMA) and connective tissue growth factor (CTGF), leading to collagen deposition. ROCK2 activation upregulates Th17 transcription via STAT3, suppresses Regulatory T cells, and regulates actin polymerization to reinforce fibrosis. Persistent cytokine secretion by Th17 maintains a vicious cycle of inflammation and fibrosis (14).

The stage-specific immune drivers of cGVHD include B-cell hyperactivity, Tfh cell expansion, and fibrosis-related factors. B-cell hyperactivity manifests as clonal proliferation and autoantibody production, with these autoantibodies targeting multiple tissue antigens to induce immune-mediated damage in target organs (15). Tfh cell expansion serves as a key driver of B cell activation: Tfh cells promote B cell differentiation and antibody production through direct cell–cell interactions and cytokine secretion (16). In terms of fibrosis, cytokines such as TGF-β and PDGF not only drive fibroblast activation and proliferation but also suppress immune cell functions, creating an immunosuppressive microenvironment that impedes normal immune regulation and further accelerates fibrotic progression (17).

## 3 Current status of multiple classification criteria for biomarkers in GVHD

Currently, the classification of GVHD biomarkers is based on multiple perspectives; however, there exists a certain degree of overlap and exclusivity among these classification methods. This article provides a review and integrative discussion of the current GVHD biomarker classification systems from the perspectives of pathophysiology, clinical application scenarios, and molecular characteristics.

### 3.1 Pathophysiological perspective

Synthesizing previous studies, from the pathophysiological perspective, biomarkers can be subdivided into three major categories: inflammation-driven, tissue damage-related, and immunoregulatory.

#### 3.1.1 Inflammation-driven biomarkers

The core function of this category of biomarkers is to mediate the inflammatory cascade in GVHD. Studies have shown that the gene expression levels of cytokines in peripheral blood mononuclear cells (PBMCs) of GVHD patients are upregulated, including IFN-γ, TNF, and interleukins (18). IL-6 and IFN-γ regulate immune cell function by activating the Janus kinase 1 (JAK1) signaling pathway (19). When the selective JAK1 inhibitor itacitinib is used in haploidentical hematopoietic stem cell transplantation (HSCT), it reduces the incidence of acute and chronic GVHD without causing severe complications—this finding has been validated in multicenter clinical trials (20). TNF-α, a key mediator of the inflammatory response, exhibits elevated serum levels in patients with aGVHD, a characteristic observed in single-center cohort studies (21). As a member of the interleukin-1 receptor family, growth-stimulated expressed gene 2 protein (ST2) plays a crucial role in inflammatory signaling pathways (22). The signaling axis formed by ST2 and interleukin-33 (IL-33) is closely associated with treatment-refractory aGVHD and non-relapse mortality (NRM) (23). Serum ST2 levels in patients with aGVHD are higher than those in control populations, and this difference has been confirmed by single-center controlled studies (24).

#### 3.1.2 Tissue damage-related biomarkers

This category of biomarkers directly reflects damage to GVHD target organs. REG3α, secreted by intestinal Paneth cells, is a specific biomarker for gastrointestinal GVHD (25). REG3α concentrations were 3-fold higher at the time of GVHD diagnosis in patients who had no response to therapy at 4 weeks than in patients who experienced a complete or partial response; patients responding to therapy still exhibited REG3α concentrations more than 3 times that of non-GVHD controls. And it can predict NRM at 4 weeks and 1 year post-transplantation—this clinical value has been confirmed in multicenter cohort studies (26). Hartwell et al. developed an innovative biomarker analysis algorithm based on single-center cohort data to evaluate blood samples collected on day 7 post-transplantation; this algorithm employs a dual-biomarker model consisting of ST2 and REG3α (27, 28). Galectin-3 (Gal-3) is a fundamental component of the galectin family (29). It induces T cell exhaustion by activating the nuclear factor of activated T cells (NFAT) signaling pathway (30), thereby alleviating tissue damage in aGVHD. The expression intensity of Gal-3 in CD4⁺ T cells is negatively correlated with intestinal pathological scores, and this association was derived from single-center clinical biopsy analyses (31). On day 15 post-haplocytotoxic HSCT, plasma Elafin levels are elevated in patients with severe cutaneous aGVHD. However, this result exhibits heterogeneity due to differences in donor characteristics and conditioning regimens, and it is only based on a single-center “discovery cohort-validation cohort” design—relevant data were obtained from single-center dual-cohort studies (32).

#### 3.1.3 Immunoregulatory biomarkers

This category of biomarkers reflects dynamic changes in the GVHD immunoregulatory system. During aGVHD onset, there is an increase in the number of effector memory T cells (TEM), a decrease in naive T cells, and enhanced proliferative activity of Treg with abnormal expression of functional markers. These dynamic changes were observed in a single-center longitudinal monitoring study at 3 months post-transplantation (33), providing evidence for immune dysregulation in aGVHD. In cGVHD, the number of follicular Tfh in peripheral blood decreases, while the number of peripheral helper T cells (Tph) increases; additionally, tissue-resident helper T cells (Trh) undergo clonal expansion in target organs. This conclusion was first mechanistically confirmed in animal models and subsequently preliminarily validated in single-center samples from patients with moderate-to-severe cGVHD, representing an integrated study combining animal models and single-center clinical correlation (34). The dynamic changes in these T cell subsets during GVHD pathogenesis profoundly reflect the disruption of the body’s immunoregulatory network, are closely associated with prognosis, and provide important evidence for GVHD treatment based on immunoregulatory mechanisms.

### 3.2 Clinical application scenario perspective

Based on the National Institutes of Health (NIH) Biomarkers, Endpoints, and Surrogate Targets (BEST) Resource, GVHD biomarkers can be further subdivided into diagnostic, predictive, response, prognostic, and risk biomarkers to meet the needs of precision medicine in different clinical scenarios (35).

#### 3.2.1 Diagnostic biomarkers

This category of biomarkers is used to confirm the presence of GVHD and involvement of target organs (3). Regulatory B cells (Bregs) exhibit significant potential in GVHD diagnosis due to their ability to maintain Treg homeostasis, promote Treg proliferation, and inhibit proinflammatory cytokine secretion (36). CD1c⁺ Bregs are induced via the PKA-CREB signaling axis. Post-HSCT, a decrease in the number of CD1c⁺ Bregs is accompanied by enhanced effector T cell activity and reduced immunosuppression, which can assist in GVHD diagnosis. Currently, evidence supporting this diagnostic potential is derived from single-center cellular function exploration studies (37). oGVHD severely impairs patients’ quality of life and visual function (38). Combined detection of IL-6, IL-10, and TNF-α improves diagnostic accuracy for oGVHD. Among these, the diagnostic value of IL-6 for oGVHD-associated dry eye has been confirmed by single-center receiver operating characteristic (ROC) curve analysis (39); further validation via a single-center small-sample correlation study of ocular surface indices demonstrated that combined detection of the three biomarkers enhances diagnostic efficacy (40). The CSF-1R inhibitor pexidartinib reduces T cell infiltration into the skin and alleviates cognitive impairment in a cGVHD mouse model, with relevant mechanisms validated in preclinical animal experiments (41). Chemokine biomarkers are a group of small-molecule cytokines or signaling proteins secreted by cells (42), and they are particularly important in GVHD diagnosis (43). Plasma levels of chemokine ligand 15 (CCL15) are elevated in cGVHD patients and correlate with NRM. This association was cross-validated using animal models and single-center human plasma samples (44), providing a new direction for cGVHD diagnosis.

#### 3.2.2 Predictive biomarkers

As measurable indicators reflecting the underlying pathophysiological processes of diseases, predictive biomarkers hold critical value in predicting disease progression and assessing dynamic evolution (45). C-X-C motif chemokine ligand 9 (CXCL9) and C-X-C motif chemokine ligand 10 (CXCL10) regulate immunopathological processes via the C-X-C chemokine receptor 3 (CXCR3). Early post-transplant serum CXCL9 levels are positively correlated with cGVHD severity, and this correlation is modulated by single-nucleotide polymorphisms (SNPs) in CXCR3 ligand genes—this finding was confirmed by multicenter cohort analysis (46). The predictive value of soluble ST2 is time-dependent: on post-transplant day 7, it can predict severe aGVHD, with an area under the curve (AUC) of 0.8125, an optimal cut-off value of 2,363 pg/mL, a sensitivity of 83.3%, and a specificity of 75.0%; on day 14, its predictive efficacy for gastrointestinal aGVHD reaches a peak, showing an AUC of 0.8007, a cut-off value of 3,419 pg/mL, a sensitivity of 81.8%, and a specificity of 82.1%; and on day 21, its predictive accuracy for overall aGVHD improves, with an AUC of 0.7092, a cut-off value of 3,464 pg/mL, a sensitivity of 65.0%, and a specificity of 80.0%. These time-dependent characteristics were derived from single-center time-series measurements. These time-dependent characteristics were derived from single-center time-series measurements (47), suggesting that dynamic monitoring is required in clinical practice to enhance predictive accuracy. Combined detection of effector CD4⁺ conventional T cells (Tconv) and CXCL9 on post-transplant day 28 can predict aGVHD, and this prediction model has been jointly validated using multicenter samples (48), providing a feasible tool for early risk stratification of aGVHD.

#### 3.2.3 Prognostic biomarkers

This category of biomarkers is used to estimate the expected disease course in patients with clinically significant conditions (49). Tumor necrosis factor receptor 1 (TNFR1), a member of the TNF receptor superfamily, is widely expressed on cell surfaces and plays important roles in anti-tumor activity and apoptosis regulation (50). Its plasma levels are elevated in aGVHD patients, and the AUC of TNFR1 at aGVHD onset is 0.71—relevant data were obtained from single-center multi-time-point detection analyses (51).

#### 3.2.4 Response biomarkers

This category of biomarkers is used to assess the efficacy of GVHD treatment. BAFF levels increase after cGVHD onset, and it enhances B-cell receptor (BCR) reactivity by upregulating NOTCH2 expression. Changes in BAFF levels can reflect treatment response in cGVHD: the mechanistic component was elucidated using animal models, and clinical relevance was established based on correlation analysis between BAFF levels and disease manifestations in single-center cGVHD patients (52). Syk inhibitors can inhibit B-cell proliferation in cGVHD patients, and BAFF levels are positively correlated with BCR signaling pathway activity, suggesting that BAFF may serve as a potential response biomarker for cGVHD treatment. This hypothesis is currently supported by single-center mechanistic exploration studies (53).

#### 3.2.5 Risk biomarkers

This category of biomarkers is used to predict the risk of GVHD development. Osteopontin (OPN) exacerbates tissue fibrosis by promoting epithelial-mesenchymal transition (EMT). Its plasma levels are upregulated in cGVHD patients, and the biomarker panel consisting of OPN, ST2, CXCL9, and matrix metalloproteinase 3 (MMP3) achieves an AUC of 0.89 for distinguishing cGVHD in the validation cohort. Its value in risk stratification has been validated in multicenter cohorts (54) (see Table 1).

**Table 1:** Classification table of current mainstream GVHD biomarkers.

Type of biomarker	Organ	Acute graft-versus-host disease		Chronic graft-versus-host disease	
		Type of biomarker	Clinical significance	Type of biomarker	Clinical significance
Diagnostic biomarker	Skins	Elafin (93)	Produced by skin keratinocytes elevate at the onset of cutaneous aGVHD	CXCL9 (94) CCL17 (95)	Exacerbates local immune responses in the skin by chemotaxis of immune cells, leading to skin tissue damage.
	Gastrointestinal tract	Reg3a (96) TIM3 (97)	Elevated concentrations in patients with intestinal aGVHD	CD34 (98)	Concentration correlates with gastrointestinal cGVHD, aids in diagnosis
	Whole body/plasma	IL2R (99) HGF (100) IL-8 (64) TNFR-1 (65) Tregs (101) Th17 (13)	Closely related to immune activation and inflammatory response.	sBAFF (102) ST2 (103) CXCL9 (54) OPN (103) CXCL10 (103) CCL19 (44) MMP 3(103)	All of these levels are influenced by a variety of factors and the combined application of aids in the diagnosis of cGVHD.
	Pulmonary	MMP3 (104)	Not yet widely used and needs to be diagnosed in combination with other markers.	/	/
Predictive biomarker	Whole body/plasma	ST2 (105) Reg3a (105)	ST2 and Reg3a levels are usually elevated during exacerbations	ST2 (103) CXCL9 (103)	Predicting treatment resistance or disease progression.
Reactive biomarker	Gastrointestinal tract	REG3α (106)	Concentration changes reflect steroid resistance	sBAFF (107) IL-10 (76)	Treatment response assessment
	Whole body/plasma	TIM3 (108) ST2 (109) TNFR1 (110) IL-2R (111) Tregs (112) Th17 (112)	Dynamic changes in their levels reflect whether T cell activation is effectively regulated or not.	TNF-α (113) ST2 (114)	Reflecting the immune inflammatory state in patients with chronic GVHD
Prognostic biomarker	Gastrointestinal tract	REG3α (115)	High concentration associated with 1-year non-recurrent mortality rate.	MMP9 (116) Reg3a (117)	Predicting disease progression.
	Whole body/plasma	ST2 (115)	14-day post-transplantation level predicts 6-month mortality.	CD163 (118) ST2 (119) CXCL9 (120)	Associated with moderate/severe cGVHD progression.
Risk biomarker	Whole body/plasma	ST2 (115) REG3α (115)	Early post-transplant elevations suggest high risk and high concentrations suggest the risk of treatment failure	Reg3α (117) CXCL10 (121) ST2 (103) MMP3 (121) CXCL9 (120) OPN (103)	Potential value in predicting the risk of developing chronic GVHD.
Novel biomarker	Whole body/plasma	MiRNA (122) Extracellular vesicles (EVs) (123)	Assessing severity and trends in aGVHD	IgG glycosylation (124) miRNA (125) EVs (126)	The different levels of immune regulation, gene expression regulation, and intercellular communication (EVs), respectively, provide new perspectives for understanding the pathological process of cGVHD.
	Gastrointestinal tract	Gut microflora (127)	Flora imbalance can further increase the risk of infection.	Gut microflora (128)	Based on the results of gut microflora testing, targeted treatments are possible.

### 3.3 Molecular characteristic perspective

#### 3.3.1 Protein biomarkers

**Figure 3:**
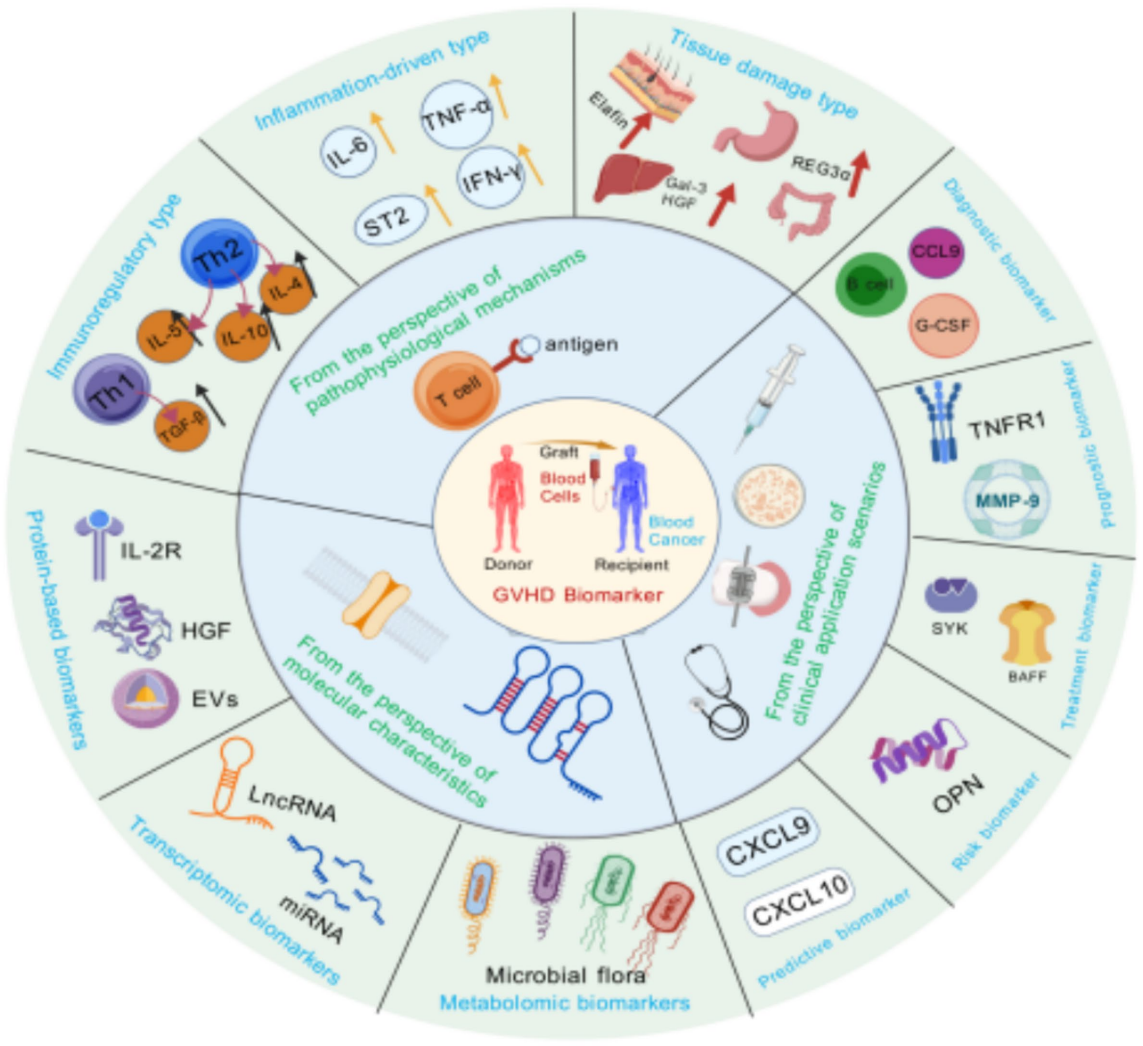
Multi-view classification of GVHD biomarkers. This figure integrates the classification of GVHD biomarkers from three perspectives: pathophysiological mechanisms, clinical scenarios, and molecular characterization. The pathophysiological mechanism perspective covers immunomodulatory, inflammation-driven, and tissue damage biomarkers; the clinical application scenario perspective is categorized into diagnostic, prognostic, response, risk, and predictive biomarkers; and the molecular characterization perspective includes metabolomics, transcriptomics, and protein biomarkers. The classification of different perspectives intersects with each other, which comprehensively demonstrates the diversity of GVHD biomarkers and helps to understand their different roles in the diagnosis and treatment of the disease. Created with BioGDP.com (10).

Protein biomarkers play an indispensable role in disease diagnosis and assessment of disease severity (55), and they can serve as potential targets for drug development (56) (Figure 3). Interleukin-2 receptor (IL-2R) is upregulated due to donor T cell activation in GVHD, and monitoring its expression levels can assist in the early diagnosis of aGVHD (57). Takehitolmado et al. conducted preclinical studies in a GVHD mouse model and found that hepatocyte growth factor (HGF) gene transfection improves mouse survival and alleviates intestinal and thymic epithelial cell damage—this protective effect is hypothesized to be associated with the anti-apoptotic biological properties of HGF (58). HGF alleviates intestinal epithelial damage via anti-apoptotic effects, and its serum levels are elevated in aGVHD patients—based on single-center small-sample serum level detection (59). Extracellular vesicles (EVs) are secreted by various cell types and play a critical role in the secretion of soluble factors such as cytokines, growth factors, chemokines, and hormones (60). They have emerged as potential novel biomarkers for multiple diseases, including aGVHD (61). Human mesenchymal stem cell-derived exosomes alleviate aGVHD by regulating the miR-16-5p/activating transcription factor 6 (ATF6)/C/EBP homologous protein (CHOP) axis—this mechanism has been confirmed by in vitro cellular experiments and animal models (62).

#### 3.3.2 Transcriptomic biomarkers

The core of this category of biomarkers is microRNAs (miRNAs)—a class of non-coding single-stranded RNA molecules encoded by endogenous genes (63). miRNA-155 is upregulated in effector T cells of aGVHD animal models (64), and inhibiting its expression reduces aGVHD severity. Relevant mechanistic exploration was conducted using preclinical animal models (65). miRNAs activate Toll-like receptors 7/8 (TLR7/8) in target cells via endocytosis, thereby inducing dendritic cell maturation and donor T cell proliferation. The conclusion that this pathway is associated with target organ damage in aGVHD was derived from pathway exploration in animal models (6).

#### 3.3.3 Metabolomic biomarkers

Metabolomic biomarkers are small-molecule metabolites produced by bodily metabolic activities, including amino acids, sugars, lipids, nucleotides, and their derivatives (66). The serum stearic acid/palmitic acid (SA/PA) ratio decreases on post-transplant day 7, which can diagnose grade II-IV aGVHD. Its efficacy in predicting aGVHD prognosis has been validated via multicenter metabolomic analysis (67), demonstrating potential for clinical application. The gut microbiota is a complex and important microecosystem in the human body, and it has been confirmed to participate in immune system development and influence host susceptibility to aGVHD (68, 69). Butyrate, a metabolite of the gut microbiota, exerts a protective effect on GVHD target organs—this protective effect was confirmed in animal models (70). In human samples, only an association between gut microbiota composition and MHC-II expression in intestinal epithelial cells has been observed, with relevant analyses derived from an integrated study combining animal models and single-center microbiota research (71).

## 4 Challenges to basic research and clinical application of GVHD biomarkers

### 4.1 Specificity and sensitivity of biomarkers

**Figure 4:**
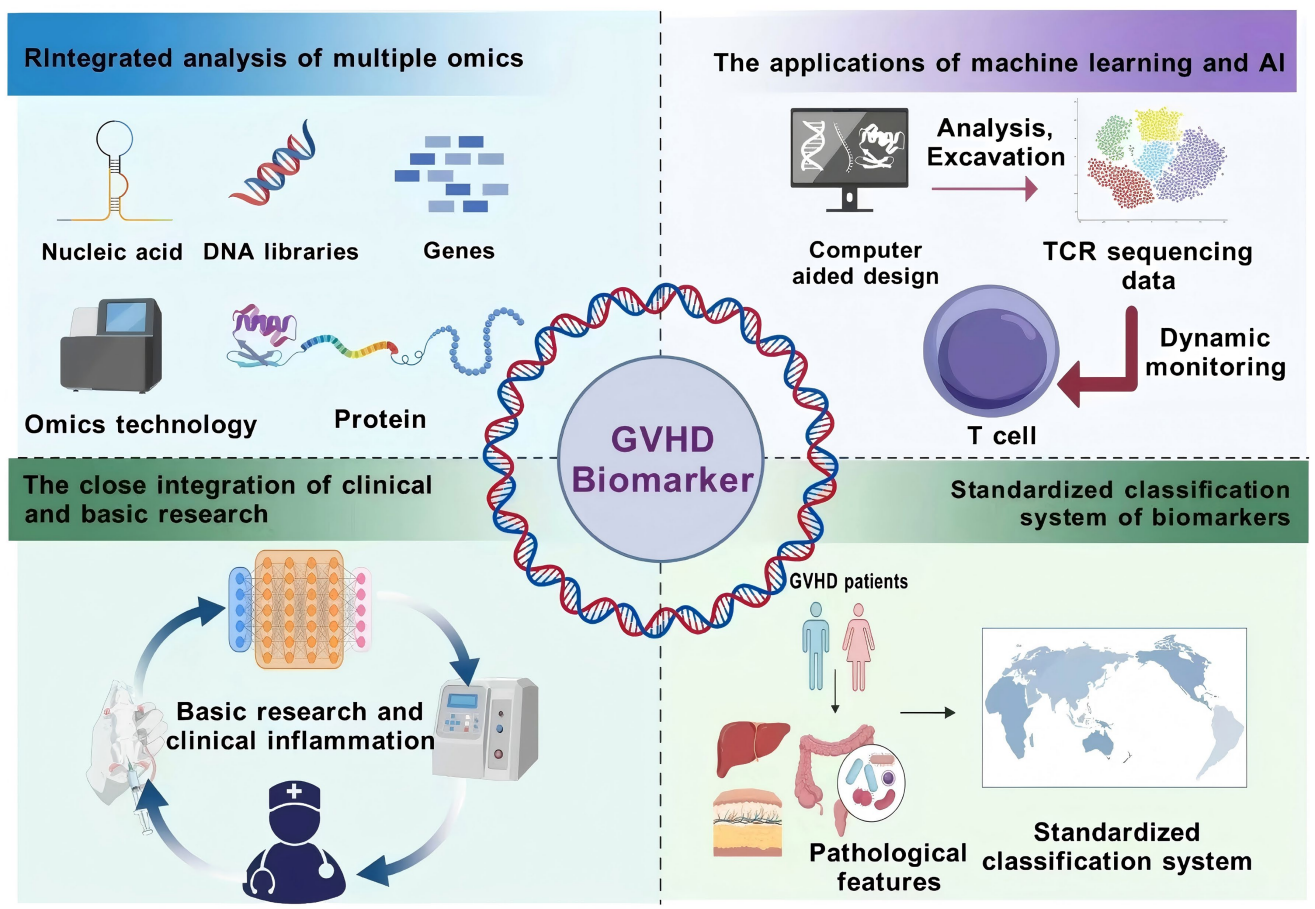
GVHD Biomarker Integration Pathway Advocacy. This figure presents the integration path of GVHD biomarkers. Multi-omics joint analysis integrates genomics, transcriptomics, and other multi-omics data to comprehensively analyze the pathogenesis; machine learning and artificial intelligence are used to analyze the biomarker data, mine potential markers, and monitor their dynamic changes; clinical and basic research are closely integrated to validate the validity and feasibility of the biomarkers and to promote the translation of the results; and a standardized classification system is established to unify the testing methods and standards and to improve the clinical application value. These paths provide a direction for solving the current challenges of biomarker research. Created with BioGDP.com (10).

Despite the identification of numerous potential biomarkers for GVHD, their specificity and sensitivity remain inadequate for clinical application (72) (Figure 4). Although TNF-α is elevated in the serum of patients with aGVHD, it is also highly expressed in other inflammatory conditions, such as post-transplant sepsis, and thus cannot be used alone to distinguish aGVHD (73). As a candidate biomarker for cutaneous aGVHD, elastase exhibits variations in plasma level cutoffs and diagnostic efficacy across different studies due to differences in donor characteristics and conditioning regimens; furthermore, the small sample sizes in these studies result in insufficient stability of the findings. Consequently, further validation and optimization of biomarker combinations are necessary to enhance the accuracy of diagnostic and prognostic evaluations.

### 4.2 Dynamics and time dependence of biomarkers

GVHD is characterized by a dynamic pathological process, where biomarker expression levels fluctuate over time.

Consequently, a single biomarker assessment may not adequately capture disease progression and treatment response, as it reflects only a specific temporal snapshot and fails to encompass the disease’s dynamic nature. The predictive value of soluble ST2 is time-dependent; relying solely on detection at a single time point easily leads to missed identification of specific organ involvement or misjudgment of disease severity. REG3α is elevated during the onset of gastrointestinal aGVHD and decreases following effective treatment. The lack of dynamic monitoring for REG3α may result in missed early intervention windows or incorrect assessment of treatment response. To more accurately assess the status of GVHD, it is imperative to establish a dynamic monitoring system capable of real-time tracking of biomarker fluctuations and generating continuous data. Integrating these data with clinical symptoms and treatment protocols for comprehensive evaluation can enhance physicians’ understanding of disease evolution (74), facilitate timely adjustments to therapeutic strategies, and ultimately improve treatment efficacy and patient prognosis.

### 4.3 Individual differences and heterogeneity of biomarkers

Significant individual differences and heterogeneity among patients with GVHD present challenges for biomarker research and its applications. Variations in immune response, genetic background, and treatment experience both prior to and following transplantation may influence biomarker expression levels (75). The degree of donor-recipient human leukocyte antigen (HLA) matching influences the expression of C-X-C motif CXCL9. Variations in conditioning regimens alter the serum levels of HGF: patients who receive total body irradiation (TBI) have higher HGF levels than those undergoing chemotherapy-based conditioning. Notably, HGF levels show no direct correlation with the incidence of aGVHD, which can easily interfere with risk assessment. Additionally, patients’ underlying diseases can increase OPN levels, weakening the correlation between OPN and the degree of fibrosis in cGVHD. Therefore, when developing personalized biomarker testing protocols, these individual differences must be fully considered.

### 4.4 Clinical validation and standardization of biomarkers

Numerous potential biomarkers for GVHD remain in the research phase and have not yet undergone extensive clinical validation (76). Although exosomes have emerged as novel biomarkers for aGVHD, their miRNA expression profiles associated with the disease have only been validated in single-center small-sample studies, and no multicenter validation has been conducted. Protein biomarkers are commonly detected using enzyme-linked immunosorbent assay (ELISA), while metabolomic biomarkers rely on mass spectrometry (MS) for detection. Differences in these technical platforms result in variations in clinical accessibility of these biomarkers. It is imperative to establish standardized testing protocols and validation procedures to enhance the clinical applicability of these biomarkers (77).

## 5 Suggested integration pathways for GVHD biomarkers

In basic research and practical clinical applications, it is not difficult to find that the three classification methods of GVHD biomarkers based on different perspectives have various limitations:

The classification of GVHD biomarkers according to pathophysiological mechanisms is grounded in scientific and theoretical principles; however, it presents certain limitations. A single marker, such as ST2, may be involved in multiple pathways, resulting in ambiguous classification (78). Various biomarker types interact and exert cross-influences (79), such as inflammatory factors impacting tissue damage and immune regulation. Focusing on a single marker while neglecting its synergistic effects and dynamic changes within the pathological process fails to fully capture the complex pathological nature of GVHD.

Biomarkers for GVHD, when considered from the perspective of clinical application scenarios, hold significant potential in the realms of diagnosis, prediction, prognosis, and the assessment of response and risk. However, they encounter challenges in practical implementation. The functionality of these biomarkers may vary throughout the disease course; initially, they may enhance immune response and disease progression, thereby aiding in diagnosis. In later stages, they may contribute to immune homeostasis or facilitate tissue repair, thus aiding in the prediction of patient survival (80), demonstrating their dual efficacy as biomarkers. For instance, the levels of BAFF may be influenced by the stage of the disease, therapeutic interventions, and other immunological factors, with its predictive value and risk assessment potentially evolving over time.

There are differences in the technology platforms required for the detection of GVHD biomarkers based on molecular characterization. For example, protein-based markers are commonly detected using ELISA (81), whereas metabolomic markers mostly rely on mass spectrometry (82). This dependence on technological platforms leads to differences in the clinical accessibility of different markers and affects their widespread use.

### 5.1 Joint multi-omics analysis

Joint multi-omics analysis involves the integration and examination of genomics, transcriptomics, proteomics, metabolomics, and other multi-omics data (83). An allo-HSCT cohort encompassing subgroups of aGVHD, cGVHD, and non-GVHD was selected, and genomic data, transcriptomic data, proteomic data, and metabolomic data were collected simultaneously from this cohort (84). After processing the data using batch correction methods, core variables were screened via LASSO regression, and a correlation network was constructed using weighted gene co-expression network analysis (WGCNA). Finally, the efficacy of the integrated panel was validated in independent multicenter cohorts to ensure its ability to distinguish between GVHD and non-GVHD, as well as to identify different target organ involvement scenarios. This work aims to further advance the clinical pilot application of the panel. This approach enables a comprehensive understanding of the pathogenesis of GVHD at various levels and facilitates the identification of additional potential biomarkers (85). Furthermore, combined multi-omics analysis can uncover the interrelationships and synergistic effects among different markers, thereby providing a theoretical foundation for the development of a standardized biomarker classification system (85).

### 5.2 Applications of machine learning and artificial intelligence

Machine learning and artificial intelligence technologies are increasingly employed in biomarker research. Through the application of machine learning algorithms, extensive biomarker datasets can be analyzed and mined to identify potential biomarkers (86). For instance, a study utilized machine learning algorithms to analyze TCR sequencing data, thereby revealing the dynamic changes in T cell clones of varying grades in GVHD patients (48). With biomarker data and clinical indicators from multicenter cohorts used as input variables, a logistic regression algorithm was employed to construct the model, which outputs GVHD development risk scores and treatment response probabilities. Following this, the model was validated in multiple independent centers, and risk score thresholds were set to guide the formulation of clear decisions during the clinical translation phase. It realizes the transformation from biomarkers to individualized treatment decisions through machine learning, providing a new paradigm for precision management of aGVHD (87).

### 5.3 Close integration of clinical and basic research

The integration of clinical and basic research is crucial for enhancing the clinical applicability of biomarkers (88). Clinical research serves to verify the validity and feasibility of biomarkers in practical settings, while basic research offers theoretical support for their discovery and application. Mechanisms of biomarkers were validated using cGVHD mouse models: BAFF regulates B cell activation, Gal-3 affects T cell cytotoxicity via the NFAT signaling pathway (88). Meanwhile, the levels of corresponding biomarkers were measured in clinical cohorts to confirm their correlation coefficients with disease activity. Furthermore, close integration of clinical and basic research facilitates the translational application of these biomarkers and accelerates the clinical translation of research findings.

### 5.4 Establishment of a standardized biomarker classification system

Establishing a standardized biomarker classification system is essential for enhancing the clinical utility of biomarkers (89). The MAGIC consortium has developed a biomarker-based classification system for evaluating the severity of aGVHD (90).

Inclusion thresholds for various types of biomarkers should be defined: for example, inflammation-driven biomarkers need to be demonstrated to have a correlation coefficient of no less than 0.5 with GVHD inflammatory indicators in at least two multicenter cohorts, while diagnostic biomarkers should have an AUC of no less than 0.75 when detected alone. Unified technical protocols for detection should be established: ELISA should be adopted for protein biomarkers, and standardized mass spectrometry parameters should be used for metabolomic biomarkers. Application guidelines should be developed based on clinical scenarios; for instance, the diagnosis of gastrointestinal aGVHD requires combined detection of REG3αand ST2. Furthermore, a standardized biomarker classification system should consider the interrelationships and synergistic effects between different biomarkers to enhance the clinical utility of these biomarkers.

## 6 Discussion on current standard protocols for the clinical application of biomarkers

Currently, despite significant progress in the discovery and validation of biomarkers, there remains a lack of widely recognized and uniformly applied standardized clinical practice guidelines for the use of biomarkers in the management of GVHD following allo-HSCT. A variety of promising biomarkers (91)—such as ST2 and REG3α for GVHD, and CXCL9 and soluble BAFF for cGVHD—have been validated in at least two independent cohorts via large-scale proteomics and reproducible detection methods. However, consensus has not been reached on key clinical parameters, including optimal detection time points, unified cut-off values, and standards for combining biomarkers with clinical indicators. This has hindered their integration into routine standardized clinical practice (92).

Currently, in clinical practice, the application of biomarkers remains primarily in the exploratory and experimental phase rather than in standardized use. For instance, patients with standard-risk aGVHD are stratified to receive treatment with sirolimus or prednisone; some centers continuously monitor ST2 to assess treatment response in steroid-refractory aGVHD. Nevertheless, such practices are limited to specific clinical trials or single-center protocols, lacking multi-institutional validation and regulatory approval (76).

Establishing standardized clinical practice guidelines for biomarker use requires prioritizing prospective, multicenter studies to address existing gaps. These studies should focus on validating well-established biomarkers to develop standardized protocols—including optimal sampling timings days 7–14 post-allo-HSCT for aGVHD risk stratification and day 100 post-allo-HSCT for cGVHD screening, clinically meaningful cut-off values, and algorithms that combine biomarkers with clinical assessments. Maintaining consistent consensus standards for biomarker identification is crucial for translating promising biomarkers into reproducible, widely applicable clinical standards. This will facilitate improved risk stratification, treatment decision-making, and post-transplant outcomes.

## 7 Conclusion

This review systematically examines the current state of research on GVHD biomarkers, including their classification systems and associated challenges. The analysis encompasses multiple dimensions, such as pathophysiological mechanisms, clinical application scenarios, and molecular properties, highlighting the fragmentation within the existing biomarker classification system and underscoring the urgent need for its integration. Furthermore, the paper identifies challenges related to specificity, sensitivity, dynamic changes, individual differences, and clinical validation in current research. It proposes feasible approaches, including multi-omics joint analysis, the application of machine learning and artificial intelligence, the integration of clinical and basic research, and the establishment of a standardized classification system.

Despite notable advancements in the investigation of GVHD biomarkers, their clinical implementation continues to encounter several obstacles. Future research should focus on the integration and analysis of multi-omics data in conjunction with machine learning and artificial intelligence technologies to enhance the specificity and sensitivity of these biomarkers. Furthermore, large-scale clinical validation and the development of standardized assays are essential for advancing the clinical application of biomarkers. A limitation of this review is that, although an integrated classification framework was proposed, specific implementation and validation data were not provided. Future research should build upon this foundation, further refine the classification criteria, and validate their efficacy through multicenter clinical studies to offer a more reliable basis for the precise diagnosis and treatment of GVHD.
